# Supplementary material for: Empirical methods for controlling false positives and estimating confidence in ChIP-Seq peaks
Source: BMC Bioinformatics. 2008 Dec 5;9:523. doi: 10.1186/1471-2105-9-523 (PMC2628906; doi:10.1186/1471-2105-9-523)
Supplement: Additional File 1 — A variety of html documents from the USeq web site detailing the available applications, their best usage, output file type descriptions, command line menus, etc. [file 1471-2105-9-523-S1.zip › USeqUserGuides/JavaDoc.zip!/JavaDoc/trans/misc/CalculateGeneExtensions.html]

CalculateGeneExtensions


|  |  |  |
| --- | --- | --- |
| **Overview** \| **Package** \| **Class** \| **Use** \| **Tree** \| **Deprecated** \| **Index** \| **Help** | |  |
| **PREV CLASS**   **NEXT CLASS** | **FRAMES**    **NO FRAMES**     **All Classes** |  |
| SUMMARY: NESTED \| FIELD \| CONSTR \| METHOD | DETAIL: FIELD \| CONSTR \| METHOD |  |


---


## trans.misc Class CalculateGeneExtensions

```
java.lang.Object
  trans.misc.CalculateGeneExtensions
```

---

``` public class CalculateGeneExtensions extends java.lang.Object ```

Finds isolated genes that are expressed and using Huber's segmentation algorithm, slices it into three and prints the extension.
Note, this is not stranded. If you want to keep it stranded then parse the UCSC Gene Table and use appropriate xxx.bar files.

---

**Constructor Summary**

`CalculateGeneExtensions(java.lang.String[] args)`


| **Method Summary** | |
| --- | --- |
| `void` | `estimateExtensions()` |
| `void` | `findExpressedGenes()`             Find genes with no neighbors, replaces the UCSCGeneLine[] array in the genes HashMap. |
| `void` | `findIsolatedGenes()`             Find genes with no neighbors, replaces the UCSCGeneLine[] array in the genes HashMap. |
| `void` | `flagSegmentedGenes()` |
| `boolean` | `intersects(UCSCGeneLine first, int start, int end)`             Assumes coordinates are inclusive. |
| `static void` | `main(java.lang.String[] args)` |
| `void` | `makeSegDats()`             Segments genes: applies median value across entire exon and intron structure, collects intensities surrounding gene, runs Hubers segmentation script on parsed gene. |
| `static void` | `printDocs()` |
| `void` | `processArgs(java.lang.String[] args)`             This method will process each argument and assign new varibles |
| `void` | `segment()` |
| `void` | `segmentGenes()` |
| `void` | `segmentGrArray(Gr[] grs)` |
| `void` | `writeTableOfIntensities()` |

**Methods inherited from class java.lang.Object**

`equals, getClass, hashCode, notify, notifyAll, toString, wait, wait, wait`

**Constructor Detail**

### CalculateGeneExtensions

```
public CalculateGeneExtensions(java.lang.String[] args)
```


**Method Detail**

### estimateExtensions

```
public void estimateExtensions()
```

---


### flagSegmentedGenes

```
public void flagSegmentedGenes()
```

---


### segmentGenes

```
public void segmentGenes()
```

---


### segment

```
public void segment()
```

---


### writeTableOfIntensities

```
public void writeTableOfIntensities()
```

---


### makeSegDats

```
public void makeSegDats()
```

:   Segments genes: applies median value across entire exon and intron structure,
    collects intensities surrounding gene,
    runs Hubers segmentation script on parsed gene.

---


### segmentGrArray

```
public void segmentGrArray(Gr[] grs)
```

---


### findExpressedGenes

```
public void findExpressedGenes()
```

:   Find genes with no neighbors, replaces the UCSCGeneLine[] array in the genes HashMap.

---


### findIsolatedGenes

```
public void findIsolatedGenes()
```

:   Find genes with no neighbors, replaces the UCSCGeneLine[] array in the genes HashMap.

---


### intersects

```
public boolean intersects(UCSCGeneLine first,
                          int start,
                          int end)
```

:   Assumes coordinates are inclusive.

---


### processArgs

```
public void processArgs(java.lang.String[] args)
```

:   This method will process each argument and assign new varibles

---


### printDocs

```
public static void printDocs()
```

---


### main

```
public static void main(java.lang.String[] args)
```


---


|  |  |  |
| --- | --- | --- |
| **Overview** \| **Package** \| **Class** \| **Use** \| **Tree** \| **Deprecated** \| **Index** \| **Help** | |  |
| **PREV CLASS**   **NEXT CLASS** | **FRAMES**    **NO FRAMES**     **All Classes** |  |
| SUMMARY: NESTED \| FIELD \| CONSTR \| METHOD | DETAIL: FIELD \| CONSTR \| METHOD |  |


---
